# Food Insecurity and Behavioral Characteristics for Academic Success in Young Adults Attending an Appalachian University

**DOI:** 10.3390/nu10030361

**Published:** 2018-03-16

**Authors:** Rebecca L. Hagedorn, Melissa D. Olfert

**Affiliations:** Davis College of Agriculture, Natural Resources, and Design, Division of Animal and Nutritional Sciences, Department of Human Nutrition and Foods, West Virginia University, 333 Agricultural Sciences Building, Laboratory G25, P.O. Box 6108, Morgantown, WV 26506-6108; USA; rlhagedorn@mix.wvu.edu

**Keywords:** food insecurity, young adult, college, student, behavior

## Abstract

In order to investigate the impact of food insecurity on college students in a highly health disparate region we (1) assessed the prevalence of food insecurity among young adults at a large, rural university in Appalachia, and (2) investigated the relationship between food insecurity and behavioral characteristics including academic performance, coping strategies, and money expenditure. A cross-sectional design was used to capture a representative sample of young adults attending a large, central Appalachian university in Fall 2016. The United States Department of Agriculture (USDA) Adult Food Security Survey was used to measure food insecurity. Independent variables include money expenditure (MES), coping strategies (CSS), academic performance (APS), and demographic, health, economic and culinary variables. Participant responses (*n* = 692) showed one third (36.6%) of respondents were food-insecure. Students with higher scores for MES and CSS had significantly higher odds of being food-insecure (odds ratio (OR) = 2.07; 95% confidence interval (CI) 1.81 to 2.38 and OR = 1.20; 95% CI 1.16 to 1.23, respectively). The odds of high APS scores (OR = 0.79; 95% CI 0.73 to 0.86) were inversely related to food insecurity. Results of the logistic regression showed MES, CSS, health, and school year remained a significant predictor of food insecurity in college students. These findings suggest behavioral differences in terms of coping strategies, money expenditure, and academic progress among food-insecure students and can be used to identify and target at-risk students to promote student food security and well-being.

## 1. Introduction

Nearly thirteen percent (15.8 million households) of Americans were food-insecure at some point in 2015 [1]. The risk of food insecurity is affected by socioeconomic status [2,3], ethnicity [4], educational attainment [5], and geographic location [6,7]. Food insecurity has been shown to be associated with inadequate diet [8,9,10,11], poor health [5,12,13], lower cognitive and academic performance [14,15,16,17,18], and higher rates of mental health and substance use disorders [12,19,20,21,22,23]. Indeed, food insecurity is related to poor physical, cognitive, and emotional health in all age populations.

Maintaining optimal health and well-being during college is especially important because it is related to academic achievement and degree attainment [15]. However, until recently, little scientific work has examined food insecurity in the young adult population attending college. Previous research on college campuses shows food insecurity prevalence is higher than the national average, with a wide range of 14–59% of the student population classified as food-insecure [24]. In addition to identifying prevalence, many of these studies examined correlates of food insecurity among the young adult population, showing food insecurity as it relates to income or financial aid status [25,26], government assistance [25,27], employment status [28], and living or housing arrangements [27,29]. 

The effect of food insecurity on college students’ behaviors and academic achievement has been minimally investigated [13,24,30]. In response to the stress of college, many students develop behavioral patterns to cope with their environment [15]. Broton and Goldrick-Rab reported that students were more likely to rely on coping behaviors such as changing eating habits, borrowing money, or postponing bill payments to make ends meet [31]. However, this study reported the percent of the student population displaying coping strategies, but failed to determine whether food-insecure students displayed these coping behaviors more often [31]. In addition to coping strategies, food-insecure students are likely to have different spending behaviors. The role of food insecurity on academic progress and student-reported behaviors is largely unknown. 

As previously stated, residing in geographically rural areas can increase the risk of being food-insecure. A systematic review of food insecurity studies on college campuses included samples from an urban southwestern university [32], urban southeastern university [26], rural western university [25], and pacific island university [29] but lacked studies of colleges or universities from the Appalachian region [33]. Appalachia is recognized for being unique from the rest of the country in terms of economic, health, and academic characteristics [34]. Specifically, in regards to higher education, while Appalachia has improved in degree attainment in recent decades, the percent of adults with bachelor degrees is still 7% below the national average [35,36]. With the suggested impact of food insecurity on educational attainment, it is important to investigate the prevalence of food insecurity among young adults attending college within Appalachia to promote degree fulfilment.

The objectives of the present study were to (1) assess the prevalence of food insecurity among young adults attending college at a large, rural university in Appalachia, and (2) investigate the relationship between food insecurity and behavioral characteristics including academic performance, coping strategies, and money expenditure. 

## 2. Materials and Methods

### 2.1. Study Design

This cross-sectional study examined a sample of young adults attending a large, Appalachian university in fall 2016, as part of a larger research project in conjunction with seven other universities in the Appalachian and Southeastern regions of the United States [37]. Participants were currently enrolled college students. All subjects gave their written informed consent for inclusion before they participated in the study. The study was conducted in accordance with the Declaration of Helsinki, and the protocol was approved by the Institutional Review Board at West Virginia University (170350219).

### 2.2. Participants and Procedures

A nonprobability sample of undergraduate and graduate students attending a large, land grant university in central Appalachia was recruited during the fall 2016 semester. All graduate and undergraduate professors teaching a fall 2016 course (across three local campuses, including 14 colleges and schools housed at the university) (*n* = 1191), were emailed an online survey link to share with enrolled students. This is an estimated 22,000 undergraduate and 6000 graduate students, although a university student listerv was not available for research access to directly contact students. Students across all disciplines and academic years were eligible to complete the survey. Interested students selected the link, taking them to Qualtrics (Qualtrics, Provo, UT, USA), an anonymous, online questionnaire platform. Participants were instructed to read the informed consent and those who accepted consent were allowed to complete the survey. Students who denied the consent were thanked for their time. Students were incentivized to complete the survey by a chance to win a $100 American Express gift card by entering their contact information following survey completion. Contact information remained separate from the results of the survey to protect participant identity. To avoid collecting data when students would more likely be provided by family support, the survey remained open from September until late November prior to when students went home for Thanksgiving break [37].

### 2.3. Survey Design

The 56-item survey was developed by an Appalachian Multistate Collaborative to investigate food insecurity in college students attending an Appalachian Higher Education Institutions. The survey, built and administered via Qualtrics, consisted of the United States Department of Agriculture Adult Food Security Screener (USDA AFSS), money expenditure scale (MES), coping strategies scale (CSS), academic progress scale (APS). MES, CSS, APS scale Cronbach’s alpha were determined as 0.7225, 0.8888, 0.6945, respectively. The remaining questions consisted of the following variable topics: demographic, economic, health, and culinary. 

*Dependent variable*: The USDA AFSS is a ten-item validated food security screener, pulled from the USDA Household Food Security Module, and is a common method for distinguishing between food secure and food-insecure individuals. The AFSS measures behaviors and conditions regarding food purchasing and intake (i.e., In the last 12 months, did you ever eat less than you felt you should because there wasn’t enough money for food?). Responses are grouped into four categories based on affirmative responses into high (no food access problems), marginal (anxiety over food situation), low (reduced diet quality and variety), and very low (reduced food intake and/or disrupted eating patterns) food security classification. 

*Independent variables*: The MES is an 8-item tool that measured how often in the past 12 months that students spent money on other items instead of using the money to purchase food [37], with never, sometimes, and often answer choices. The items assessed for monetary purchases included substance purchases (i.e., alcohol, cigarettes, and recreational drugs), transportation (i.e., public transportation fees, car repairs, and gasoline), pet care, and tattoos. 

The CSS development was guided by previous food insecurity literature and used in previous college settings [37,38,39,40]. The 29-item scale examined how often students used coping strategies in the past 12 months with never, sometimes, and often answer choices. The coping topics included saving, support, food intake/access and selling. Support questions asked if students took fewer classes, used less utilities, shared housing and food responsibilities with others, planned or stretched meals, used coupons, or saved on medications or medical appointments. Support questions included if students participated in a research study/clinical trial to buy food, borrowed money from family or friends, attended functions with free food or where you “pay when you can”, obtained food from a food bank, food pantry or assistance program (e.g., Supplemental Nutrition Assistance Program (SNAP), Women, Infants and Children (WIC), etc.), visited family on weekends to bring back food to school, held one or more part/full time jobs or used a credit card to buy food. Questions on food intake/access asked if students ate more than normal when food was plentiful, took food home from on-campus dining hall, ate less healthy meals to eat more food, purchased processed food, obtained food from a dumpster or trash, or bartered services/items to buy food. Lastly, the selling questions enquired if students ever sold textbooks, personal possessions, blood/plasma or sperm/eggs to obtain food. 

Academic behaviors were captured using the 4-item APS, on which students reported their perceived academic performance. Students completed questions regarding class attendance and attention span, understanding the concepts taught in class, and progression towards graduating on time (i.e., How would you rate your class attendance?). Grade point average (GPA) was also self-reported by students as an indicator of academic progress but assessed separately from the APS. 

Demographic variables included gender, home region (e.g., Midwest, Northeast, etc.), age, marital status, ethnicity, dependents, student status, school year, housing, car ownership, and utilization of public transportation. Economic variables included receiving financial aid, employment status, and purchase of a meal plan. Health variables included self-reported health status, having health insurance and body mass index (BMI) (calculated from self-reported height and weight). Also included were two questions with a culinary focus regarding how often students cooked for themselves and how they would rate their cooking skills. 

### 2.4. Statistical Analysis

Descriptive statistics were computed for all demographic, economic, health, and culinary variables as appropriate. BMI was calculated from self-reported height and weight, and categorized using the World Health Organization (WHO) BMI classification [41]. Food security status was determined for the 10 AFSS questions in accordance with the Guide to Measuring Household Food Security scoring system [42]. As protocol states, zero affirmative answers reflected high food security, 1–2 marginal food security, 3–5 low food security, and 6–10 very low food security. Prevalence of food insecurity was determined by combining those who scored in the high or marginal food secure categories (food secure) and those who scored in the low and very low food secure categories (food-insecure). 

The MES and CSS were scored on a 3-point scale with 1 point representing “never”, 2 points to the “sometimes,” and 3 points to the “often” responses. Total scores for MES could range from 8 to 24 points and CSS scores could range from 29 to 87 points. The 4-item APS was scored on a 4-point scale with 4 points for the “excellent,” 3 for the “good,” 2 for the “fair,” and 1 for the “poor” responses. Therefore, scores on the APS could range from 4 to 16 points. All scales were left continuous for analysis, with higher MES scores representing more spending on items before buying food, higher CSS scores representing more reliance on coping strategies to acquire and maintain food sources, and higher APS scores representing a more positive perception of academic behaviors. 

Pearson Chi-square analyses were used to determine bivariate associations of food secure and food-insecure students with sociodemographic and behavioral variables. MES, CSS, APS, GPA and BMI were assessed as a continuous variables and Wilcoxon analysis was used due to lack of normality to compare means of food-insecure and food secure students. Simple logistic regression was used to predict food security status from scores on MES, APS, and CSS scales. Forward selection multivariate logistic regression was used in a full model to predict food insecurity from the all significant or close to significant categorical and continuous variables from Chi-square and Wilcoxon analyses. Lack of fit was assessed by Hosmer and Lemeshow Goodness-of-Fit test (*χ*^2^(8) = 9,17, *p* = 0.3278) indicating the model was adequate. 

Data were analyzed using JMP and SAS software (JMP^®^, Version Pro 12.2, SAS Institute Inc., Cary, NC, USA, 2015; SAS^®^, Version 9.4, SAS Institute Inc., Cary, NC, USA, 2002–2012). Significance criterion alpha for all tests was 0.05.

## 3. Results

The survey was completed by 716 undergraduate and graduate students during the fall 2016 semester. As food insecurity was the primary outcome, participants who did not supply a full response to the ten questions USDA AFSS (*n* = 24) were excluded from analysis. A final sample of 692 was used for data analysis. 

Analysis of the AFSS scores showed 439 respondents (63.4%) as food secure comprised of 236 highly food secure (34.1%) and 203 marginally food secure (29.3%) respondents. The remaining 253 respondents (36.6%) were classified as food-insecure consisting of 115 with low food security (16.6%) and 138 with very low food security (20.0%). 

Respondents were predominately white (87.3%), single (94.3%), females (71.0%) with average age 21.3 years ± 4.0 standard deviation (SD). Students were spread across all academic years with the majority being full time (97.55) with an average GPA of 3.4 ± 0.45. Most students lived off campus (67.9%) and owned a car (71.5%) yet many still relied on public transportation (63.4%). Student economic situations varied with majority having one or more part-time jobs (44.6%), receiving financial aid (80.4%), and not having a student meal plan (67.9%). Health status of students was predominately high with 85.0% reporting excellent or good health and 98.3% having health insurance. Student BMI varied from 14.9 to 52.6 (Mean 25.0 ± 5.3) and most respondents fell in the healthy (18.5–24.9) BMI range (56.7%) followed in prevalence by the overweight (25–29.9) category (23.2%). 

**Figure 1:**
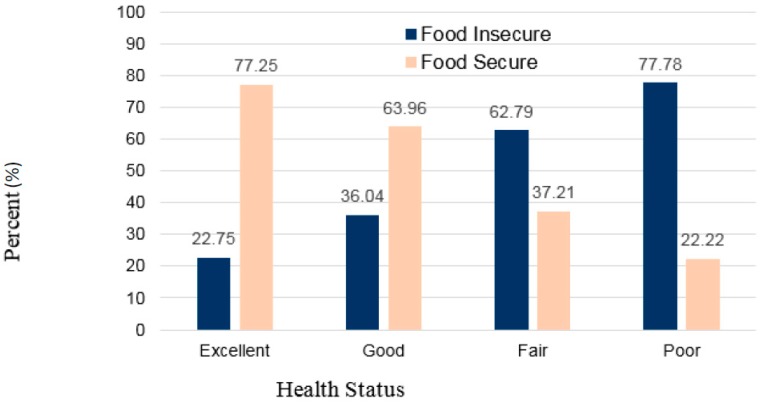
Percent of health status category by food security status among students. Association of health status and food security group showed a higher proportion of food-insecure students reported poor or fair health while food secure students reported good or excellent health. Chi-square (*p* < 0.0001).

Sample characteristics by food security status are presented in Table 1. Investigation of categorical sociodemographic variables with food security showed significant associations between food security status and academic year (*p* = 0.0130), self-reported health status (*p* < 0.0001), and housing (*p* = 0.0269). Specifically, food insecurity was associated with academic year and found to be at the highest prevalence during the sophomore (46.0%) and junior (45.8%) years with the lowest prevalence in graduate students (29.4%). Students who lived off campus displayed higher prevalence of food insecurity (36.9%) compared to those who lived on campus (30.5%). Self-reported health status showed a higher proportion of food-insecure students who reported fair or poor health represented in Figure 1.

Mean BMI was not significantly different between food secure and food-insecure students (*p* = 0.2636), however, BMI classification showed association that trended toward significance (*p* = 0.0601), with higher prevalence of obese classification in the food-insecure population than in the food secure population. Food insecurity status also showed significant differences in GPA as average GPA of food-insecure students was 3.33 ± 0.03 and average GPA of food secure students was 3.51 ± 0.02 (*p* < 0.0001). 

Significant relationships were found between food security status and MES, CSS, and APS scores (*p* < 0.0001 for all). Students who reported spending money on other items before purchasing food, as represented by high MES scores (odds ratio (OR) = 2.07; 95% CI 1.81–2.38) and displayed more coping strategies for food had significantly higher odds of being food-insecure (OR = 2.07; 95% CI 1.81–2.38 and OR = 1.20; 95% CI 1.16–1.23, respectively). The odds of high academic progress scores (OR = 0.79; 95% CI 0.73–0.86) were inversely related to food insecurity. 

All variables significant in simple analyses (MES, CSS, GPA, APS, school year, housing and health) and close to significant (BMI category) were entered in a full logistic regression model. Forward selection was used to identify the most important variables predictive of food insecurity. MES (OR = 1.44; 95% CI 1.24–1.67), CSS (OR = 1.17; 95% CI 1.13–1.23), school year (specifically freshman vs. graduate student, OR = 2.85; 95% CI 1.36–5.97) and health (OR = 2.88; 95% CI 1.54–5.41) remained significant predictors of food insecurity. MES and CSS were the best predictors of food insecurity based on *p*-values of Wald Chi-Square (data not shown) [43]. Results are shown in Table 2. 

## 4. Discussion

To our knowledge, this study is the first to investigate the prevalence of food insecurity at a central Appalachian university and the second within the region all together. Along with McArthur et al. [37], this study provides a representation of food insecurity correlates in young adults attending a large Appalachian university and the relationships between food security and behavior (money expenditure, coping strategies and academic progress). Over one third of students (36.6%) were food-insecure, with higher prevalence of food insecurity occurring in sophomore and junior year students, those who live off campus, and those reporting poor health. Food-insecure students displayed behaviors that differed from food secure students including spending more money on other items, engaging in more coping strategies to find food, and having lower academic success in the classroom.

The prevalence of food insecurity found in this study is consistent with previous studies that have determined food insecurity rates among college students are higher than the national average [1,33]. Studies show food insecurity rates ranging from 14% to 59% at universities with varying demographic locations and sample characteristics [33]. Within the Appalachian region, McArthur et al. [37], found a higher prevalence of food insecurity at 46.2% of student population, suggesting the increased need within the region. 

Associations between food security and insecurity with covariates is consistent with some previous findings. The health of food-insecure students has been previously reported as being fair or poor when compared to food secure students, comparable with our results [25,27,37,44]. This could be attributed to the role access to food and dietary quality play on mental and physical heath [3,11,12,20,23]. Additionally, our study found that academic year of the student influenced food insecurity, with increased food-insecure populations occurring following the freshman year, similar to previous research [37,45,46]. Housing status has been conflicted in previous literature on influence on food insecurity, with some studies finding it plays a significant role, and others showing no differences in food security status by housing status [25,29,47]. This study found that housing, specifically living off campus, influenced the prevalence of food insecurity. The influence of academic year and housing are especially important as avenues for food insecurity interventions in at-risk populations. Additionally, both housing and academic year were more common in McArthur et al. [37], making them potential variables of interest throughout the Appalachian region. 

Beyond correlates, this study investigated the money expenditure and coping behaviors used by university attending young adults. Students who spent more money on items such as substances or rent instead of food (higher MES score) were at higher odds of being food-insecure. There are possible explanations for this finding. First, many college students are new to financial independence and lack the skills necessary to manage money efficiently. This in turn could lead to deprioritizing food and, ultimately, to developing food insecurity. More specifically, with the limited income of many college students, it is possible that food and financial management skills can aid in the prevention of food insecurity [26]. Secondly, the increase in the cost of university tuition and decrease in subsidies for students may play a role in the spending habits of students and consequently lead to food insecurity. In this study, food-insecure students commonly displayed behavioral coping strategies to make ends meet and obtain food. This is consistent with previous studies showing college students often cut back on activities, changed eating habits, borrowed money, and even forwent purchasing school supplies as coping strategies in order to afford food [31]. The impact of coping on student success is equivocal with some, but not all, studies finding a relationship between use of coping strategies and academic success [48,49]. Similar to our results, one study found reliance on coping strategies in college students as a predictor of academic achievement [15]. 

In the present study, academic achievement was assessed by the APS score which enumerates how the student rated their own overall progress in school including graduating on time, class attendance, attention span in class, and understanding of concepts taught in class. Food-insecure students displayed greater odds of receiving lower APS scores and lower GPA, representing poorer academic success. Food insecurity has been associated with increased behavioral problems and emotional burdens that can impact a student’s success in academia [14]. In particular in the college population, food-insecure students are less likely to attend and perform well in class and more likely to withdraw from a course all together [30,50]. Other studies confirm this association through GPA and have found that students with a GPA above 3.1 were 60% less likely to be food-insecure [25] with another reporting food-insecure students having a mean GPA of 3.1 vs. 3.4 in food secure students [51]. 

This cross-sectional study has limitations that must be noted. First, the use of a non-probability sample from a single geographical, predominately Caucasian public university prevents generalizability to university populations such as universities outside the Appalachian region, community colleges, or private institutions, and those with ethnic diversity. Although respondents were disproportionally white, this is representative of demographics in the Appalachian region compared to other regions in the United States and can be interpreted as such [52]. Additionally, the cross-sectional design and non-probability sample cannot set establishment of causation. Next, the self-report of measures may limit the validity of results and the inclusion of freshman may provide inconstancy within literature. McArthur et al. [37] excluded freshman from their sample due to the AFSS question referencing the previous 12 months. This has occurred within literature but is not consistent across studies within college students, therefore our sample included freshman based on the studies such as Bruening et al. [33]. Lastly, as a listerv for students was unavailable it is unknown how many students were exposed to the study and depict an accurate response rate. The response received is approximately 2.5% of the total student body, however the demographic characteristics collected are consistent with reports from the university on student body characteristics. 

In conclusion, this study sheds light on the prevalence of food insecurity among young adults attending a large university in central Appalachia. Further, the study reveals the impact food insecurity can have on students’ behaviors with increased money expenditure and coping strategies, and decreased academic progress in food-insecure students. The behaviors of young adult college students are essential for success and degree retention, with numerous students leaving college without successful degree completion, causing a financial burden to both the university and the student [50]. Providing for the basic needs of students and fostering positive behaviors would promote student success and are important avenues for addressing food insecurity on college campuses. University administrators and public health experts can benefit from this information through targeted interventions for promoting academic success. 

## Figures and Tables

**Table 1:** Characteristics of respondents and correlations with food security status.

Variable	Food Secure		Food-Insecure		*p*-Value
	*n*	%	*n*	%	
Total Population					
	438	63.4	253	36.6	
Gender					
Male	120	28.7	70	28.7	0.9957
Female	298	71.3	174	71.3	
Ethnicity					
African American	9	2.3	10	4.3	0.2640
Asian	15	3.8	3	1.3	
Hispanic	11	2.8	6	2.6	
White	349	87.3	203	87.5	
Other/Multiracial	16	4.0	10	4.3	
Marital Status					
Single	394	93.8	233	95.1	0.4885
Married	26	6.2	12	4.9	
Dependents					
Has Dependents	8	1.9	7	2.9	0.4250
No Dependents	412	98.1	238	97.1	
School Year					
Freshman	106	25.6	48	19.9	0.0130 *
Sophomore	47	16.6	40	11.4	
Junior	66	15.9	55	22.8	
Senior	97	23.4	58	24.1	
Graduate Student	98	23.7	40	16.6	
Home Region					
Midwest	34	8.1	14	5.7	0.3006
Northeast	126	30.0	80	32.7	
Southeast	246	58.6	148	60.4	
Southwest	5	1.2	0	0	
West	9	2.1	3	1.2	
Car Ownership					
Yes	286	71.5	166	71.6	0.9889
No	114	28.5	66	28.5	
Use Public Transportation					
Yes	260	65.0	141	60.8	0.2878
No	140	35.0	91	39.2	
Housing					
On Campus	141	35.3	62	26.7	0.0269 *
Off Campus	259	64.8	170	73.3	
Employment					
Unemployed	169	42.3	93	40.1	0.1509
Part-time Job	172	43.0	110	47.4	
Full-time Job	25	6.3	19	8.2	
Other	34	8.5	10	4.3	
Financial Aid					
Yes	324	81.0	184	79.3	0.6062
No	76	19.0	48	20.7	
Meal Plan					
Yes	136	34.0	67	28.9	0.1839
No	264	66.0	165	71.1	
Health Status					
Excellent	130	32.5	38	16.4	<0.0001 *
Good	236	59.0	133	57.3	
Fair	32	8.0	54	23.3	
Poor	2	0.5	7	3.0	
Health Insurance					
Yes	395	98.8	226	97.4	0.2157
No	5	1.25	6	2.6	
BMI Category					
Underweight	16	3.9	11	4.6	0.0601 ^†^
Normal	243	58.7	128	53.3	
Overweight	101	24.4	51	21.3	
Obese	54	13.0	50	20.8	
Cook for Self					
Often	192	48.0	96	41.4	0.1804
Sometimes	150	37.5	104	44.8	
Never	58	14.5	32	13.8	
Cooking Skills					
Excellent	105	26.3	47	20.3	0.3710
Good	190	47.5	121	52.2	
Fair	85	21.3	50	21.6	
Poor	20	5.0	14	6.0	
	Mean	SD	Mean	SD	
BMI	24.72	0.24	25.57	0.39	0.2638
Age	21.43	0.21	21.06	0.23	0.8116
GPA	3.51	0.02	3.33	0.03	<0.0001 *
MES Score	10.58	0.06	12.33	0.14	<0.0001 *
CSS Score	36.72	0.32	46.61	0.50	<0.0001 *
APS Score	13.28	0.09	12.39	0.13	<0.0001 *

Demographic data represented in frequency and percentages. Pearson Chi-square frequency and Wilcoxon analyses were performed. * *p* < 0.05, ^†^ *p* < 0.07. SD, standard deviation; BMI, body mass index; GPA, grade point average; MES, money expenditure scale; CSS, coping strategies scale; APS, academic progress scale.

**Table 2:** Logistic regression model predicting food insecurity in students.

Variable	Odds Ratio	95% Confidence Interval
MES Score	1.44	1.24–1.67
CSS Score	1.17	1.13–1.22
School Year		
Freshman	2.85	1.36–5.97
Sophomore	2.23	0.99–5.07
Junior	1.94	0.95–3.96
Senior	1.75	0.88–3.47
Health		
Excellent/Good vs. Fair/Poor	2.88	1.54–5.41
GPA	0.65	0.40–1.06

Selection criteria for the model entry was *p* < 0.07. Variables from simple analyses were entered into a forward selection multiple logistic regression model. MES, money expenditure scale; CSS, coping strategies scale; school year and health remained significant predictors of food security status. GPA, grade point average was not a significant predictor.

## References

[1] Coleman-Jensen A., Rabbitt M.P., Gregory C., Singh A. (2016). Household Food Security in the United States in 2015.

[2] Cook J.T., Frank D.A. (2008). Food security, poverty, and human development in the United States. Ann. N. Y. Acad. Sci..

[3] Bocquier A., Vieux F., Lioret S., Dubuisson C., Caillavet F., Darmon N. (2015). Socio-economic characteristics, living conditions and diet quality are associated with food insecurity in France. Public Health Nutr..

[4] Coleman-Jensen A., Gregory C., Singh A. (2014). Household Food Security in the United States in 2013.

[5] Seligman H.K., Laraia B.A., Kushel M.B. (2010). Food insecurity is associated with chronic disease among low-income NHANES participants. J. Nutr..

[6] America F. (2017). Rural Hunger Facts.

[7] Haldemen L. (2006). A Comparison of Food Insecurity Levels and Weight Status among Rural-and Urban-Residing Latinos/Hispanics in North Carolina. http://srdc.msstate.edu/ridge/projects/recipients/05_haldeman_final.pdf.

[8] Champagne C.M., Casey P.H., Connell C.L., Stuff J.E., Gossett J.M., Harsha D.W., McCabe-Sellers B., Robbins J.M., Simpson P.M., Weber J.L. (2007). Poverty and food intake in rural America: Diet quality is lower in food-insecure adults in the Mississippi Delta. J. Am. Diet. Assoc..

[9] Robaina K.A., Martin K.S. (2013). Food insecurity, poor diet quality, and obesity among food pantry participants in Hartford, CT. J. Nutr. Educ. Behav..

[10] Rose D. (1999). Economic determinants and dietary consequences of food insecurity in the United States. J. Nutr..

[11] Mello J.A., Gans K.M., Risica P.M., Kirtania U., Strolla L.O., Fournier L. (2010). How is food insecurity associated with dietary behaviors? An analysis with low-income, ethnically diverse participants in a nutrition intervention study. J. Am. Diet. Assoc..

[12] Tarasuk V., Mitchell A., McLaren L., McIntyre L. (2013). Chronic physical and mental health conditions among adults may increase vulnerability to household food insecurity. J. Nutr..

[13] Farahbakhsh J., Hanbazaza M., Ball G.D., Farmer A.P., Maximova K., Willows N.D. (2017). Food-insecure student clients of a university-based food bank have compromised health, dietary intake and academic quality. Nutr. Diet..

[14] Alaimo K., Olson C.M., Frongillo E.A. (2001). Food insufficiency and American school-aged children's cognitive, academic, and psychosocial development. Pediatrics.

[15] DeBerard M.S., Spielmans G., Julka D. (2004). Predictors of academic achievement and retention among college freshmen: A longitudinal study. Coll. Stud. J..

[16] Jyoti D.F., Frongillo E.A., Jones S.J. (2005). Food insecurity affects school children’s academic performance, weight gain, and social skills. J. Nutr..

[17] Perez-Escamilla R., Pinheiro de Toledo Vianna R. (2012). Food insecurity and the behavioral and intellectual development of children: A review of the evidence. J. Appl. Res. Child. Inf. Policy Child. Risk.

[18] Shankar P., Chung R., Frank D.A. (2017). Association of food insecurity with children’s behavioral, emotional, and academic outcomes: A systematic review. J. Dev. Behav. Pediatr..

[19] McLaughlin K.A., Green J.G., Alegría M., Costello E.J., Gruber M.J., Sampson N.A., Kessler R.C. (2012). Food insecurity and mental disorders in a national sample of US adolescents. J. Am. Acad. Child. Adolesc. Psychiatry.

[20] Weaver L.J., Hadley C. (2009). Moving beyond hunger and nutrition: A systematic review of the evidence linking food insecurity and mental health in developing countries. Ecol. Food Nutr..

[21] Weinreb L., Wehler C., Perloff J., Scott R., Hosmer D., Sagor L., Gundersen C. (2002). Hunger: Its impact on children’s health and mental health. Pediatrics.

[22] Davison K.M., Holloway C., Gondara L., Hatcher A.S. (2018). Independent associations and effect modification between lifetime substance use and recent mood disorder diagnosis with household food insecurity. PLoS ONE.

[23] Davison K.M., Gondara L., Kaplan B.J. (2017). Food Insecurity, Poor Diet Quality, and Suboptimal Intakes of Folate and Iron Are Independently Associated with Perceived Mental Health in Canadian Adults. Nutrients.

[24] Cady C.L. (2014). Food insecurity as a student issue. J. Coll. Character.

[25] Patton-Lopez M.M., Lopez-Cevallos D.F., Cancel-Tirado D.I., Vazquez L. (2014). Prevalence and correlates of food insecurity among students attending a midsize rural university in Oregon. J. Nutr. Educ. Behav..

[26] Gaines A., Robb C.A., Knol L.L., Sickler S. (2014). Examining the role of financial factors, resources and skills in predicting food security status among college students. Int. J. Consum. Stud..

[27] Hughes R., Serebryanikova I., Donaldson K., Leveritt M. (2011). Student food insecurity: The skeleton in the university closet. Nutr. Diet..

[28] Freudenberg N., Manzo L., Jones H., Kwan A., Tsui E., Gagnon M. (2011). Food Insecurity at CUNY: Results from a Survey of CUNY Undergraduate Students.

[29] Chaparro M.P., Zaghloul S.S., Holck P., Dobbs J. (2009). Food insecurity prevalence among college students at the University of Hawai’i at Mānoa. Public Health Nutr..

[30] Silva M.R., Kleinert W.L., Sheppard A.V., Cantrell K.A., Freeman-Coppadge D.J., Tsoy E., Roberts T., Pearrow M. (2015). The Relationship between Food Security, Housing Stability, and School Performance Among College Students in an Urban University. J. Coll. Stud. Retent. Res Theory Pract..

[31] Broton K., Goldrick-Rab S. (2016). The dark side of college (un) affordability: Food and housing insecurity in higher education. Chang. Mag. High. Learn..

[32] Bruening M., Brennhofer S., van Woerden I., Todd M., Laska M. (2016). Factors Related to the High Rates of Food Insecurity among Diverse, Urban College Freshmen. J. Acad. Nutr. Diet..

[33] Bruening M., Argo K., Payne-Sturges D., Laska M.N. (2017). The Struggle Is Real: A Systematic Review of Food Insecurity on Postsecondary Education Campuses. J. Acad. Nutr. Diet..

[34] Couto R.A. (2012). Appalachian Health and Well-Being.

[35] Appalachian Regional Commission Education—High School and College Completion Rates, 2011–2015. https://www.arc.gov/reports/custom_report.asp?REPORT_ID=68.

[36] Shaw T.C., DeYoung A.J., Rademacher E.W. (2004). Educational attainment in Appalachia: Growing with the nation, but challenges remain. J. Appalach. Stud..

[37] McArthur L.H., Ball L., Danek A.C., Holbert D. (2017). A High Prevalence of Food Insecurity Among University Students in Appalachia Reflects a Need for Educational Interventions and Policy Advocacy. J. Nutr. Educ. Behav..

[38] Kempson K., Keenan D.P., Sadani P.S., Adler A. (2003). Maintaining food sufficiency: Coping strategies identified by limited-resource individuals versus nutrition educators. J. Nutr. Educ. Behav..

[39] Pinard C., Smith T.M., Calloway E.E., Fricke H.E., Bertmann F.M., Yaroch A.L. (2016). Auxiliary measures to assess factors related to food insecurity: Preliminary testing and baseline characteristics of newly designed hunger-coping scales. Prev. Med. Rep..

[40] Maxwell D.G. (1996). Measuring food insecurity: The frequency and severity of “coping strategies”. Food Policy.

[41] World Health Organization (2000). Obesity: Preventing and Managing the Global Epidemic.

[42] Bickel G., Nord M., Price C., Hamilton W., Cook J. (2000). Guide to Measuring Household Food Security. Revised: 2000.

[43] Thompson D. (2009). Ranking Predictors in Logistic Regression.

[44] Knol L.L., Robb C.A., McKinley E.M., Wood M. (2017). Food Insecurity, Self-rated Health, and Obesity among College Students. Am. J. Health Educ..

[45] Martinez S., Maynard K., Ritchie L. (2016). Student Food Access and Security Study.

[46] Hughes R. (2012). Food insecurity amongst university students. Nutridate.

[47] Morris L.M., Smith S., Davis J., Null D.B. (2016). The prevalence of food security and insecurity among Illinois university students. J. Nutr. Educ. Behav..

[48] Ryland E.B., Riordan R.J., Brack G. (1994). Selected characteristics of high-risk students and their enrollment persistence. J. Coll. Stud. Dev..

[49] Brown N., Cross E. (1997). Coping resources and family environment for female engineering students. Coll. Stud. J..

[50] Goldrick-Rab S., Richardson J., Hernandez A. (2017). Hungry and Homeless in College: Results from a National Study of Basic Needs Insecurity in Higher Education.

[51] Martinez S., Brown E., Ritchie L. (2016). What Factors Increase Risk for Food Insecurity among College Students?. J. Nutr. Educ. Behav..

[52] Pollard K., Jacobsen L.A. (2011). The Appalachian Region in 2010: A Census Data Overview Chartbook. http://www.prb.org/pdf12/appalachia-census-chartbook-2011.pdf.

